# Risk Factors for Severe Coronavirus Disease 2019 Among Human Immunodeficiency Virus-Infected and -Uninfected Individuals in South Africa, April 2020–March 2022: Data From Sentinel Surveillance

**DOI:** 10.1093/ofid/ofac578

**Published:** 2022-11-02

**Authors:** Sibongile Walaza, Stefano Tempia, Anne von Gottberg, Nicole Wolter, Jinal N Bhiman, Amelia Buys, Daniel Amoako, Fahima Moosa, Mignon du Plessis, Jocelyn Moyes, Meredith L McMorrow, Halima Dawood, Ebrahim Variava, Gary Reubenson, Jeremy Nel, Heather J Zar, Mvuyo Makhasi, Susan Meiring, Vanessa Quan, Cheryl Cohen

**Affiliations:** Centre for Respiratory Diseases and Meningitis, National Institute for Communicable Diseases of the National Health Laboratory Service, Johannesburg, South Africa; School of Public Health, Faculty of Health Sciences, University of the Witwatersrand, Johannesburg, South Africa; Centre for Respiratory Diseases and Meningitis, National Institute for Communicable Diseases of the National Health Laboratory Service, Johannesburg, South Africa; School of Public Health, Faculty of Health Sciences, University of the Witwatersrand, Johannesburg, South Africa; MassGenics, Atlanta, Georgia, USA; Influenza Program, Centers for Disease Control and Prevention, Pretoria, South Africa; Centre for Respiratory Diseases and Meningitis, National Institute for Communicable Diseases of the National Health Laboratory Service, Johannesburg, South Africa; School of Pathology, Faculty of Health Sciences, University of the Witwatersrand, Johannesburg, South Africa; Centre for Respiratory Diseases and Meningitis, National Institute for Communicable Diseases of the National Health Laboratory Service, Johannesburg, South Africa; School of Pathology, Faculty of Health Sciences, University of the Witwatersrand, Johannesburg, South Africa; Centre for Respiratory Diseases and Meningitis, National Institute for Communicable Diseases of the National Health Laboratory Service, Johannesburg, South Africa; School of Pathology, Faculty of Health Sciences, University of the Witwatersrand, Johannesburg, South Africa; Centre for Respiratory Diseases and Meningitis, National Institute for Communicable Diseases of the National Health Laboratory Service, Johannesburg, South Africa; Centre for Respiratory Diseases and Meningitis, National Institute for Communicable Diseases of the National Health Laboratory Service, Johannesburg, South Africa; Centre for Respiratory Diseases and Meningitis, National Institute for Communicable Diseases of the National Health Laboratory Service, Johannesburg, South Africa; School of Pathology, Faculty of Health Sciences, University of the Witwatersrand, Johannesburg, South Africa; Centre for Respiratory Diseases and Meningitis, National Institute for Communicable Diseases of the National Health Laboratory Service, Johannesburg, South Africa; School of Pathology, Faculty of Health Sciences, University of the Witwatersrand, Johannesburg, South Africa; Centre for Respiratory Diseases and Meningitis, National Institute for Communicable Diseases of the National Health Laboratory Service, Johannesburg, South Africa; School of Public Health, Faculty of Health Sciences, University of the Witwatersrand, Johannesburg, South Africa; Influenza Program, National Center for Immunization and Respiratory Diseases, Centers for Disease Control and Prevention, Atlanta, Georgia, USA; Influenza Program, Centers for Disease Control and Prevention, Pretoria, South Africa; Department of Medicine, Greys Hospital, Pietermaritzburg, South Africa; Caprisa, University of KwaZulu - Natal, Pietermaritzburg, South Africa; Department of Medicine, Klerksdorp-Tshepong Hospital Complex, Klerksdorp, South Africa; Department of Medicine, Faculty of Health Sciences, University of the Witwatersrand, Johannesburg, South Africa; Department of Paediatrics and Child Health, Faculty of Health Sciences, University of the Witwatersrand, Rahima Moosa Mother and Child Hospital, Johannesburg, South Africa; Department of Medicine, Faculty of Health Sciences, University of the Witwatersrand, Johannesburg, South Africa; Department of Paediatrics, Red Cross War Memorial Hospital, and South African-Medical Research Council on Child and Adolescent Health, University of Cape Town, South Africa; Centre for Respiratory Diseases and Meningitis, National Institute for Communicable Diseases of the National Health Laboratory Service, Johannesburg, South Africa; School of Public Health, Faculty of Health Sciences, University of the Witwatersrand, Johannesburg, South Africa; Divison of Public Health Surveillance and Response, National Institute for Communicable Diseases of the National Health Laboratory Service, Johannesburg, South Africa; Divison of Public Health Surveillance and Response, National Institute for Communicable Diseases of the National Health Laboratory Service, Johannesburg, South Africa; Centre for Respiratory Diseases and Meningitis, National Institute for Communicable Diseases of the National Health Laboratory Service, Johannesburg, South Africa; School of Public Health, Faculty of Health Sciences, University of the Witwatersrand, Johannesburg, South Africa

**Keywords:** COVID-19, HIV, severity‌, South Africa

## Abstract

**Background:**

Data on risk factors for coronavirus disease 2019 (COVID-19)-associated hospitalization and mortality in high human immunodeficiency virus (HIV) prevalence settings are limited.

**Methods:**

Using existing syndromic surveillance programs for influenza-like-illness and severe respiratory illness at sentinel sites in South Africa, we identified factors associated with COVID-19 hospitalization and mortality.

**Results:**

From April 2020 through March 2022, severe acute respiratory syndrome coronavirus 2 was detected in 24.0% (660 of 2746) of outpatient and 32.5% (2282 of 7025) of inpatient cases. Factors associated with COVID-19-associated hospitalization included the following: older age (25–44 [adjusted odds ratio {aOR}= 1.8, 95% confidence interval (CI) = 1.1–2.9], 45–64 [aOR = 6.8, 95% CI = 4.2–11.0] and ≥65 years [aOR = 26.6, 95% CI = 14.4–49.1] vs 15–24 years); black race (aOR, 3.3; 95% CI, 2.2–5.0); obesity (aOR, 2.3; 95% CI, 1.4–3.9); asthma (aOR, 3.5; 95% CI, 1.4–8.9); diabetes mellitus (aOR, 5.3; 95% CI, 3.1–9.3); HIV with CD4 ≥200/mm^3^ (aOR, 1.5; 95% CI, 1.1–2.2) and CD4 <200/mm^3^ (aOR, 10.5; 95% CI, 5.1–21.6) or tuberculosis (aOR, 12.8; 95% CI, 2.8–58.5). Infection with Beta (aOR, 0.5; 95% CI, .3–.7) vs Delta variant and being fully vaccinated (aOR, 0.1; 95% CI, .1–.3) were less associated with COVID-19 hospitalization. In-hospital mortality was increased in older age (45–64 years [aOR, 2.2; 95% CI, 1.6–3.2] and ≥65 years [aOR, 4.0; 95% CI, 2.8–5.8] vs 25–44 years) and male sex (aOR, 1.3; 95% CI, 1.0–1.6) and was lower in Omicron-infected (aOR, 0.3; 95% CI, .2–.6) vs Delta-infected individuals.

**Conclusions:**

Active syndromic surveillance encompassing clinical, laboratory, and genomic data identified setting-specific risk factors associated with COVID-19 severity that will inform prioritization of COVID-19 vaccine distribution. Elderly people with tuberculosis or people with HIV, especially severely immunosuppressed, should be prioritized for vaccination.

## Teaser

Omicron variant was associated with reduced risk of mortality and Beta associated with decreased risk of hospitalization. Syndromic surveillance can be used to describe epidemic timing, epidemiological characteristics of cases, early detection of variants, and how these impact disease severity/outcomes.

Since its emergence in December 2019, coronavirus disease 2019 (COVID-19), caused by severe acute respiratory syndrome coronavirus 2 (SARS-CoV-2), has spread globally causing severe morbidity and mortality [1]. South Africa reported its first case of laboratory-confirmed COVID-19 on March 5, 2020 and experienced 4 epidemic waves during the study period [2]. By the end of November 2021, a high proportion of the South African population had some level of SARS-CoV-2 immunity. Seroprevalence in the catchment area of 2 surveillance sites ranged from 60%–70%; however, it was estimated that <10% of cases were clinically diagnosed [3]. Observational studies, mostly conducted in high-income countries, have described risk factors associated with increased risk of severe COVID-19 (hospitalization and death), which included, older age, male sex, and presence of comorbidities [4–6]. However, data on risk factors for COVID-19-associated hospitalization and mortality in settings with high human immunodeficiency virus (HIV) or tuberculosis prevalence are limited. In South Africa in 2021, HIV prevalence was 19.5% among individuals aged 15–49 years, whereas tuberculosis prevalence was among the highest globally [7].

As SARS-CoV-2 evolves and new variants and lineages emerge, monitoring the impact they have on disease severity remains important. Sentinel surveillance programs established to monitor influenza and other respiratory pathogens represent a potentially important platform for monitoring SARS-CoV-2 in low- and middle-income countries, like South Africa, where resources for testing may be limited. South Africa's vaccine program began on February 17, 2021, and as of April 7, 2022, 44% of individuals aged ≥18 years were fully vaccinated against SARS-CoV-2 (1 dose of COVID-19 vaccine Jansen) or 2 doses of Pfizer-BioNTech COVID-19 Vaccine Comirnaty) [8], booster doses of vaccine were introduced from December 24, 2021 with subsequent additional doses added over time (additional information in the Supplementary material) [9, 10].

Using a well established syndromic surveillance program for influenza-like-illness (ILI) [11] and severe respiratory illness (SRI) [12–14], we aimed to describe clinical and epidemiological characteristics of persons with laboratory-confirmed COVID-19 and identify factors associated with COVID-19 hospitalization or mortality.

## METHODS

### Surveillance Program

We enhanced our existing syndromic surveillance program for influenza, respiratory syncytial virus (RSV), and *Bordetella pertussis* to include molecular testing and genomic surveillance for SARS-CoV-2 from April 1, 2020. We conducted active, prospective, clinic-based surveillance for ILI at 4 public health clinics in 3 provinces. In addition, we conducted active prospective surveillance for SRI at 8 public sector hospitals in 5 provinces; 4 hospitals were in the same catchment areas as the ILI clinic sites.

### Case Definitions for Enrollment

Individuals aged ≥15 years presenting to outpatient surveillance clinics meeting the ILI or suspected COVID-19 surveillance case definitions including any of the following: (1) ILI—fever (≥38°C and/or self-reported fever) and cough and symptoms ≤10 days; (2) suspected COVID-19—acute (≤14 days) respiratory tract illness or other clinical illness compatible with COVID-19 (cough, sore throat, shortness of breath, anosmia, or dysgeusia) or physician-diagnosed suspected COVID-19.

Individuals aged ≥15 years hospitalized at surveillance hospitals meeting the SRI case definition including any of the following: individuals with physician-diagnosed lower respiratory tract illness (LRTI) or physician-diagnosed suspected COVID-19.

Henceforth, all outpatients meeting the case definitions used at ILI sites will be referred to as ILI cases, and all hospitalized patients meeting any of the case definitions used at SRI sites will be referred to as SRI cases.

### Study Procedures

The procedures for surveillance have been described previously [3–6]. In brief, all patients presenting at ILI sites from Monday to Friday (8:00 Am–4:00 Pm) or admitted at SRI sites from 5:00 Pm on Sunday through 1:00 Pm on Friday and meeting surveillance case definitions were eligible for enrollment. In addition, cases admitted at SRI sites over the weekend and testing positive for SARS-CoV-2 from testing conducted as part of clinical care were also enrolled if they met surveillance case definitions. Dedicated surveillance staff (surveillance officers [nurses] and research assistants) screened all outpatients or medical admissions, sought consent, and completed enrollment procedures if eligibility criteria were met. Enrolled hospitalized patients were followed until outcome (discharge, transfer, or death). Case record forms were completed through structured interviews and by reviewing hospital medical records.

### Laboratory Procedures

Nasopharyngeal swabs (Copan Italia, Brescia, Italy) were collected from study participants on enrollment, placed in universal transport medium, and stored at 4°C–8°C for transport on ice in a cooler box for testing at the National Institute for Communicable Disease (NICD) within 72 hours of collection. Nucleic acids were extracted from 200 µL transport medium using a MagNA Pure 96 automated extractor and MP96 DNA and Viral NA Small Volume v2.0 extraction kit (Roche Diagnostics, Mannheim, Germany). Extracts were tested for influenza viruses, RSV, and SARS-CoV-2 using real-time, reverse-transcription polymerase chain reaction (rRT-PCR). In addition, SARS-CoV-2-positive samples were tested by variant PCR and/or sequenced at NICD to ascertain their lineage/clade [17]. Additional information on rRT-PCR testing and sequencing methods are included in Supplementary material.

Human immunodeficiency virus testing was ordered by attending clinicians as part of the standard of care. Pretest counseling and bedside HIV testing was performed by surveillance staff for consenting patients not tested by the attending clinician. Among people with HIV (PWH), no or mild HIV immune compromise was defined as CD4^+^ T lymphocytes ≥200/mm^3^ and severe immune compromise as CD4^+^ T- ymphocytes <200/mm^3^.

### Statistical Analysis

To assess factors associated with SARS-CoV-2-hospitalization, only sites in provinces with ILI and SRI surveillance in the same catchment area were included in the analysis. All 8 hospitals were included in the analysis of factors associated with SARS-CoV-2 infection and mortality. Logistic regression was used to assess factors associated with (1) SARS-CoV-2 infection among ILI or SRI cases at all surveillance sites; (2) COVID-19 hospitalization by comparing the characteristics of SARS-CoV-2-positive SRI cases (cases) with those of SARS-CoV-2-positive ILI cases (comparison group); and (3) in-hospital mortality among SARS-CoV-2-positive patients with SRI.

The wave variable, defined as national weekly case incidence of ≥30 cases/100 000 (in-wave) and incidence <30 cases/100 000 (out of wave), and period variable, defined as time in months since the first documented case in South Africa to time of presentation, were included a priori in all models, to account for bias due to possible changes in enrollment of cases and clinical management during periods of increased transmission and changes in severity as population immunity increases as a result of infection or vaccination over time and improved treatment becomes available. Vaccination status was also included a priori as an important confounder on infection and severity. Fully vaccinated was defined as 1 dose of COVID-19 vaccine Jansen or 2 doses of Pfizer-BioNTech COVID-19 Vaccine Comirnaty.

We hypothesized that differences exist in patient demographics, clinical presentation, underlying conditions, province, whether admitted during wave and time of admission since first case in South Africa, vaccination status, and duration of hospitalization between COVID-19 cases who were hospitalized or died and those not hospitalized or did not die. Variable selection was based on significance from the univariable logistic regression analysis (*P* < .05), the correlation between indicators and completeness of data. To account for clustering by site, random effects were applied in all multivariable logistic regression models. Significant variables *P* < .10 on univariate analysis were evaluated for inclusion in the multivariable models. Nonsignificant variables at *P* ≥ .05 were dropped using manual backward elimination. Pairwise interactions were assessed by inclusion of product terms for all variables remaining in the final multivariable additive model. Stata version 14 (StataCorp Limited, College Station, TX) was used for analysis.

### Ethical Approval

The ILI and SRI protocols were approved by the University of Witwatersrand Human Research Ethics Committee (HREC), reference M180832 and M140824, respectively. Additional approvals were received from other HRECs (Supplemental Material). This activity was reviewed by the Centers for Disease Control and Prevention (CDC) and was conducted consistent with applicable US federal law and CDC policy (see eg, 45 C.F.R. part 46, 21 C.F.R. part 56; 42 U.S.C. 241(d); 5 U.S.C. 552a; 44 U.S.C. 3501 et seq).

### Patient Consent Statement

Written consent was obtained from participants aged ≥18 years and from parent/guardian of participants aged <18 years. In addition to consent from parent/guardian, an assent was obtained from participants aged 7–17 years.

## RESULTS

From April 1, 2020 to March 31, 2022, 10 040 patients aged ≥15 years were enrolled from all sentinel sites, 9781 (97.4%) of whom had SARS-CoV-2 results. Of the cases with results, 2757 (28.2%) had ILI and 7025 (71.8%) had SRI. Among ILI cases, 10 (0.4%) were referred to hospital after consultation at an ILI site and were excluded from further analysis. A total of 9771 patients were included in the analysis for description of factors associated with testing positive for SARS-CoV-2 (Table 1).

**Table 1.** Demographic and Clinical Characteristics of Patients With and Without Laboratory-Confirmed (rRT-PCR) SARS-CoV-2 Presenting With ILI or Hospitalized With SRI at Sentinel Sites in South Africa, April 2020–March 2022 (*N* = 9771)

ILI = 2746					SRI, *n* = 7025			
Characteristic	SARS-CoV-2 Negative, *n*/*N* (%)	SARS-CoV-2 PositiVe, *n*/*N* (%)	Odds Ratio (95% CI)	aOR (95% CI)	SARS-CoV-2 Negative, *n*/*N* (%)	SARS-CoV-2 Positive, *n*/*N* (%)	Odds Ratio (95% CI)	aOR (95% CI)
Age Group								
15–24 y	351/2086 (16.8)	94/660 (14.2)	Reference	Reference	278/4743 (5.9)	77/2282 (3.4)	Reference	Reference
25–44 y	1154/2086 (55.3)	354/660 (53.6)	1.1 (0.9–1.5)	1.2 (.9–1.6)	1875/4743 (39.5)	643/2282 (28.1)	1.2 (.9–1.6)	1.2 (.9–1.7)
45–64 y	504/2086 (24.2)	180/660 (27.3)	1.3 (1.0–1.8)	1.4 (1.1–1.9)	1622/4743 (34.2)	973/2282 (42.6)	2.1 (1.6–2.8)	1.6 (1.1–2.1)
≥65 y	77/2086 (3.7)	32/660 (4.9)	1.6 (1.0–2.6)	1.9 (1.2–3.1)	968/4743 (20.4)	589/2282 (25.8)	2.3 (1.7–3.0	1.2 (.9–1.7)
Black vs other race	1481/2085 (71.0)	491/659 (74.5)	0.8 (.6–1.1)	…	4076/4735 (86.1)	1852/2279 (81.3)	0.7 (.6–.8)	…
Male sex	973/2084 (46.7)	268/658 (40.7)	0.8 (.6–.9)	0.8 (.7–.9)	2237/4736 (47.2)	871/2277 (38.3)	0.7 (.6–.8)	0.7 (.6–.8)
Province								
Gauteng	N/A	N/A	N/A	N/A	1467/4743 (30.9)	569/2282 (24.9)	1.1 (1.0–1.3)	1.4 (1.1–1.7)
KwaZulu-Natal	454/2086 (21.7)	139/660 (21.06)	1.4 (1.1–1.8)	1.3 (1.0–1.7)	1088/4743 (23.0)	545/2282 (23.9)	1.5 (1.2–1.7)	1.4 (1.0–1.7)
Mpumalanga	N/A	N/A	N/A	N/A	810/4743 (17.1)	276/2282 (12.1)	Reference	Reference
North West	1041/2086 (49.9)	392/660 (59.4)	1.7 (1.4–2.2)	1.6 (1.3–2.0)	773/4743 (16.3)	509/2282 (22.3)	1.9 (1.6–2.3)	2.3 (1.8–2.9)
Western Cape	591/2086 (29.1)	129/660 (19.6)	Reference	Reference	605/4743 (12.8)	378/2282 (16.9)	1.9 (1.5–2.3	1.9 (1.5–2.3)
Months since start of pandemic to consultation	…	…	1.0 (1.0–1.0)	1.0 (1.0–1.1)	…	…	1.00 (1.0–1.0)	1.0 (1.0–1.0)
In wave vs out of wave	1116/2086 (53.5)	527/660 (79.9)	3.4 (2.7–4.2)	3.5 (2.8–4.4)	2499/4743 (52.7)	1941/2282 (85.1)	5.1 (4.5–5.8)	4.6 (4.0–5.3)
Underlying Medical Conditions								
Asthma	72/2083 (3.5)	9/660 (1.4)	0.4 (.2 –.8)	0.4 (.2–.8)	267/4722 (5.7)	83/2276 (3.7)	0.6 (.5–.8)	0.6 (.4–.7)
Any TB* vs no TB	20/2086 (1.0)	2/660 (0.3)	0.3 (.1–1.5)	…	822/4743 (17.3)	81/2282 (3.6)	0.2 (.1–.2)	0.2 (.1–.3)
Diabetes Mellitus	62/2082 (3.0)	17/660 (2.6)	0.9 (.5–1.6)	…	546/4724 (11.6)	478/2276 (21.0)	2.0 (1.8–2.3)	1.4 (1.2–1.7)
Obesity (BMI ≥30)	60/2083 (2.8)	28/660 (4.2)	1.5 (1.0–2.4)	…	242/4724 (5.1)	242/2275 (10.6)	2.2 (1.9–2.7)	1.4 (1.1–1.8)
HIV infected vs noninfected	406/2025 (20.1)	125/643 (19.4)	0.9 (.7–1.1)	…	2106/4461 (47.2)	570/2104 (27.3)	0.4 (.4–.5)	0.7 (.5–.7)
Hypertension^a^	217/1632 (13.3)	91/529 (17.2)	1.3 (1.0–1.7)	…	1064/3622 (29.4)	768/1836 (41.8)	1.7 (1.5–1.9)	…
Other underlying condition^b^	136/2086 (6.5)	39/660 (5.9)	0.9 (.6–1.4)	…	1039/4743 (21.9)	429/2282 (18.8)	0.8 (.7–1.0)	0.7 (.6–.9)
Pregnant**	9/910 (1.0)	3/297 (1.0)	1.0 (.3–3.9)	…	171/1321 (12.9)	167/605 (27.6)	3.0 (2.3–3.9)	…
RSV coinfection	47/2083 (2.3)	3/659 (0.5)	0.2 (.1–.6)	0.2 (.1–.6)	54/4603 (1.2)	9/2213 (.4)	0.3 (.2–.7)	0.4 (.2–.9)
COVID-19 vaccination status								
Not vaccinated/partially vaccinated	1866/2086 (89.4)	597/660 (90.1)	Reference	Reference	4315/4743 (91.0)	2187/2282 (95.8)	Reference	Reference
Fully vaccinated	158/2086 (7.6)	49/660 (7.4)	0.9 (.7–1.3)	0.9 (.6–1.3)	273/4743 (5.8)	69/2282 (3.0)	0.5 (.4–.7)	0.4 (.3–.6
Unknown	62/2086 (3.0)	14/660 (2.1)	0.7 (.4–1.3)	1.6 (.9–3.0)	155/4743 (3.3)	26/2282 (1.1)	0.2 (.2–.5)	0.8 (.5–1.3)
Duration of Symptoms (d)								
≥5 vs 0–4	686/2086 (32.9)	265/660 (40.2)	1.3 (1.1–1.5)	…	2579/4743 (54.4)	1165/2282 (51.1)	0.9 (.8–1.0)	…
Clinical management								
Supplemental oxygen administered	N/A	N/A	N/A	N/A	3044/4708 (64.7)	1728/2269 (76.2)	1.7 (1.5–1.9)	1.4 (1.2–1.6)
ICU admission	N/A	N/A	N/A	N/A	16/4708 (0.3)	47/2269 (2.1)	6.0 (3.4–10.6)	6.9 (3.4–14.0)
Duration of Hospitalization (d)								
<5days vs ≥ 5 d	…	…	…	…	1765/4668 (37.8)	1009/2252 (44.8)	1.4 (1.2–1.5)	1.2 (1.1–1.4)
In-hospital outcome								
Died	N/A	N/A	N/A	N/A	556/4687 (11.9)	387/2255 (17.2)	1.6 (1.4–1.8)	1.5 (1.2–1.8)

Abbreviations: aOR, adjusted odds ratio; CI, confidence interval; COVID-19, coronavirus disease 2019; d, days; HIV, human immunodeficiency virus; ILI, influenza-like illness; ICU, intensive care unit; N/A, not applicable; RSV, respiratory syncytial virus; rRT-PCR, real-time, reverse-transcription polymerase chain reaction; SARS-CoV-2, severe acute respiratory syndrome coronavirus 2; SRI, severe respiratory illness; TB, tuberculosis; y, years.

a Hypertension was not collected as a standalone variable; patients reported it under other underlying conditions.

b Evaluated underlying medical conditions included the following: asplenia or sickle cell anemia; chronic illness including lung, renal, liver or cardiac disease; other immunocompromising conditions (excluding HIV), including organ transplant, primary immunodeficiency, immunotherapy, and malignancy; neurological disorders and burns.

*Any TB = TB at any time (past and current).

**Evaluated among women of childbearing age (15 years–49 years).

***Vaccination of healthcare workers (phase 1 of the vaccine program) with a single dose of the COVID-19 vaccine Jansen started on February 17, 2021 and phase 2 including the general public started on May 17, 2021. Full vaccination = 1 dose of COVID-19 vaccine Jansen and 2 doses of Pfizer-BioNTech COVID-19 Vaccine Comirnaty.

**Figure 1.**
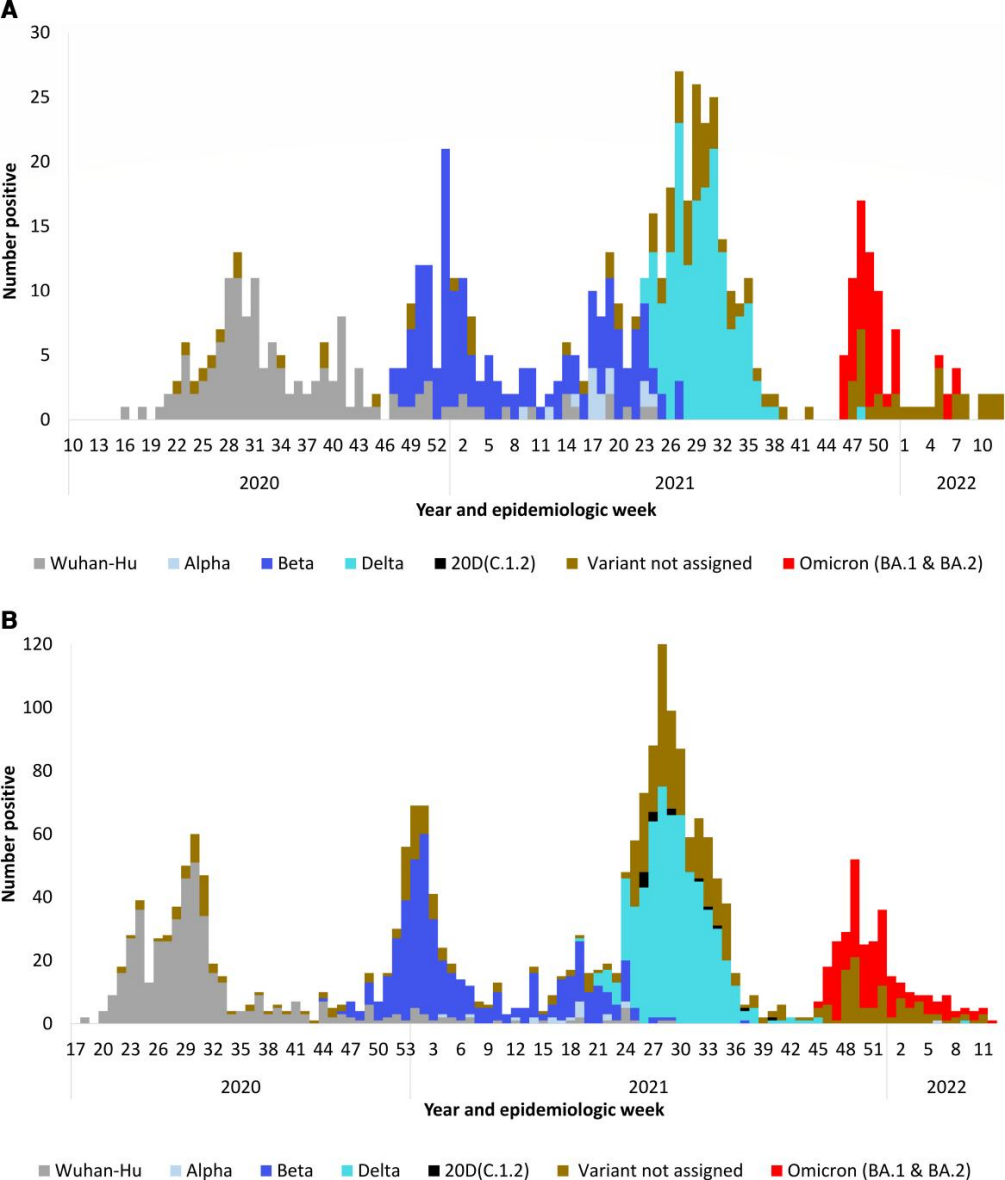
(*A*) Number of laboratory-confirmed (real-time, reverse-transcription polymerase chain reaction [rRT-PCR]) coronavirus disease 2019 (COVID-19) cases aged ≥15 years by severe acute respiratory syndrome coronavirus 2 (SARS-CoV-2) variant type and epidemiologic week, influenza-like illness surveillance, South Africa, April 2020–March 2022. (*B*) Number of laboratory-confirmed (rRT-PCR) COVID-19 cases aged ≥15 years by SARS-CoV-2 variant type and epidemiologic week, severe respiratory illness surveillance, South Africa, April 2020–March 2022.

Severe acute respiratory syndrome coronavirus 2 was detected in 24.0% (660 of 2746) of ILI and 32.5% (2282 of 7025) of SRI cases. During the study period, the COVID-19 epidemic in South Africa had 4 waves: predominated by SARS-CoV-2 Wuhan-Hu, Beta, Delta, and Omicron (BA.1 and BA.2) variants, respectively (Figure 1*A*and*B*).

The majority of ILI cases were of Black race (71.9%, 1972 of 2744) and 54.7% (1501 of 2742) were female. Human immunodeficiency virus prevalence among ILI cases was 19.9% (531 of 2668) and 85.7% (438 of 511) of PWH were on antiretroviral therapy. Eight percent (207 of 2670) of ILI cases with known COVID-19 vaccination status were fully vaccinated at the time of enrollment. Of these, 25% (118 of 207) received COVID-19 vaccine Jansen. On multivariable analysis among individuals with ILI, SARS-CoV-2-positive cases were more likely to be in older age groups (45–64 years [adjusted odds ratio {aOR}, 1.4; 95% confidence interval {CI}, 1.1–1.9] and ≥65 years [aOR, 1.9; 95% CI, 1.1–3.1]) compared to 15–24 years to present during the wave (aOR, 3.4; 95% CI, 2.7–4.2) compared to out of wave and to present ≥5 (aOR, 1.3; 95% CI, 1.1–1.5) days from date of symptom onset compared to <5 days. The SARS-CoV-2 cases were less likely to be coinfected with RSV (aOR, 0.2; 95% CI, .1–.6) and to have asthma (aOR, 0.4; 95% CI, .2–.8).

The majority of SRI cases were of Black race (81.3%, 5928 of 7014) and 55.7% (3905 of 7013) were female. The HIV prevalence among SRI cases was 40.8% (2676 of 6565) and 67.2% (833 of 2535) of PWH were on antiretroviral therapy. Five percent (342 of 6884) of cases with known COVID-19 vaccination status were fully vaccinated. Of these, 36.0% (123 of 342) received COVID-19 vaccine Jansen. On multivariable analysis, among individuals with SRI (including all 8 surveillance hospitals), SARS-CoV-2-positive cases were more likely to be 45–64 years (aOR, 1.5; 95% CI, 1.1–2.1) compared with 15–24 years, to be diabetic (aOR, 1.4; 95% CI, 1.2–1.7) or obese (aOR, 1.4; 95% CI, 1.2–1.7), to be enrolled during a wave compared to out of wave (aOR, 4.6; 95% CI, 4.0–5.3), to receive oxygen therapy (aOR, 1.4; 95% CI, 1.2–1.6), to be admitted to an intensive care unit (aOR, 6.8; 95% CI, 3.5–13.2), and to die (aOR, 1.5; 95% CI, 1.2–1.7) during admission. The SARS-CoV-2 cases were less likely to be male (aOR, 0.7; 95% CI, .6–.8), to be infected with HIV (aOR, 0.6; 95% CI, .5–.7), to have asthma (aOR, 0.6; 95% CI, .4–.7), or to have previous or current tuberculosis (aOR, 0.2; 95% CI, .1–.3) compared to those testing SARS-CoV-2 negative.

### Risk Factors for Severe Acute Respiratory Syndrome Coronavirus 2-Associated Hospitalization

From April 1, 2020 to March 31, 2022, a total of 2746 ILI and 3905 SRI cases were enrolled at the 3 sentinel sites (Western Cape, North West, and KwaZulu-Natal) with surveillance for SRI and ILI in the same catchment population. Severe acute respiratory syndrome coronavirus 2 was detected in 24.0% (660 of 2746) and 36.9% (1439 of 3905) of ILI and SRI cases, respectively. Human immunodeficiency virus prevalence was 19.4% (125 of 643) and 25.0% (345 of 1378) among SARS-CoV-2-positive ILI and SRI cases, respectively (Table 2).

**Table 2.** Risk Factors for COVID-19-Associated Hospitalization Among Persons Aged ≥15 Years at 3 Sentinel Sites^a^ in South Africa, April 2020–March 2022,^b^*N* = 6651

Characteristic	COVID-19-Associated ILI, *n*/*N* (%)	COVID-19-Associated SRI, *n*/*N* (%)	Odds Ratio (95% CI)	aOR (95% CI)
Demographic characteristics				
Age Group	…	…	…	…
15–24 y	94/660 (14.2)	51/1439 (3.5)	Reference	Reference
25–44 y	354/660 (53.6)	382/1439 (26.6)	1.9 (1.3–2.9)	1.8 (1.1–2.9)
45–64 y	180/660 (26.9)	641/1439 (44.5)	6.5 (4.4–9.7)	6.8 (4.2–11.0)
≥65 y	32/660 (4.9)	365/1439 (25.4)	19.6 (11.8–32.5)	26.6 (14.4–49.1)
Male sex	268/658 (40.7)	528/1435 (36.8)	0.8 (.7–1.0)	…
Black vs other races	491/659 (74.5)	1157/1437 (80.5)	2.3 (1.7–3.2)	2.3 (2.2–5.0)
Province	…	…	…	…
Western Cape	129/660 (19.6)	385/1439 (26.8)	2.3 (1.8–2.9)	4.0 (2.6–6.0)
North West	392/660 (59.4)	509/1439 (35.4)	Reference	Reference
KwaZulu-Natal	139/660 (21.1)	545/1439 (37.87)	3.0 (2.4–3.8)	2.6 (1.9–3.5)
Months since start of pandemic to enrollment	…	…	1.0 (1.0–1.0)	1.0 (.9–1.1)
In wave vs out of wave	527/660 (79.9)	1243/1439 (86.4)	1.3 (1.0–1.7)	1.1 (.8–1.6)
Variant				
Wuhan-Hu	136/620 (21.9)	295/1311 (22.5)	09 (.7–1.2)	1.0 (.5–1.9)
Alpha	14/620 (2.3)	11/1311 (0.8)	0.5 (.2–1.1)	0.7 (.2–1.9)
Beta	155/620 (25.0)	221/1311(16.9)	0.6 (.5–.8)	0.5 (.3–.7)
Delta	162/620 (26.1)	415/1311 (31.7)	Reference	Reference
20D (C.1.2)	1/620 (0.2)	7/1311 (0.5)	3.1 (.4–25.9)	1.7 (.2–15.2)
Omicron (BA.1 and BA.2)	55/620 (8.9)	122/1311 (9.3)	0.8 (.5–1.1)	1.1 (.6–1.8)
Variant not assigned	97/620 (15.7)	240/1311 (18.3)	1.0 (.7–1.4)	1.3 (.9–1.9)
Underlying Medical Conditions				
Asthma	9/660 (1.4)	57/1435 (4.0)	2.7 (1.3–5.6)	3.5 (1.4–8.9)
Diabetes	17/660 (2.6)	329/1435 (22.9)	9.8 (5.9–16.1)	5.3 (3.1–9.3)
Obesity (BMI ≥30)	28/660 (4.2)	161/1434 (11.2)	2.5 (1.6–3.7)	2.3 (1.4–3.9)
HIV Status				
Negative	518/600 (86.3)	1033/1299 (79.5)	Reference	Reference
Positive (no/mild immune compromise)^e^	71/600 (11.8)	161/1299 (12.39)	1.2 (.9–1.6)	1.5 (1.1–2.1)
Positive (severe immune compromise)^e^	11/600 (1.8)	105/1299 (8.1)	5.6 (3.0–10.7)	10.5 (5.1–21.6)
Hypertension^c^	91/529 (17.2)	535/1184 (45.2)	4.0 (3.1–5.2)	…
Other underlying condition^d^	99/643 (15.4)	412/1410 (29.2)	3.1 (2.4–4.1)	3.6 (2.4–5.6)
Any TB* vs no TB	2/660 (0.3)	50/1439 (3.5)	11.4 (2.7–47.2)	12.8 (2.8–58.5)
RSV infection	3/659 (0.5)	3/1377 (0.2)	0.6 (.1–3.3)	…
Pregnant**	11/297 (3.7)	79/358 (22.1)	8.0 (4.1–15.4)	…
COVID-19 Vaccination Status***				
Not vaccinated/partially vaccinated	597/660 (90.4)	1372/1439 (95.3)	Reference	Reference
Fully vaccinated	49/660 (7.4)	48/1439 (3.3)	0.3 (.2–.5)	0.1 (.1–.3)
Unknown	14/660 (2.1)	19/1439 (1.3)	0.7 (.3–1.4)	0.7 (.3–1.9)
Duration of Symptoms (d)				
0–4 vs ≥5	395/660 (59.9)	730/1439 (50.7)	1.5 (1.2–1.8)	…

Abbreviations: aOR, adjusted odds ratio; BMI, body mass index; CI, confidence interval; COVID-19, coronavirus disease 2019; HIV, human immunodeficiency virus; ILI, influenza-like illness; ICU, intensive care unit; RSV, respiratory syncytial virus; SRI, severe respiratory illness; TB, tuberculosis; y, years.

a ILI sites = Jouberton clinic in North West, Gateway clinic in KwaZulu-Natal, and Eastridge and Mitchell's Plain clinics in Western Cape, SRI = Klerksdorp-Tshepong hospital complex in North West, Edendale Hospital in KwaZulu-Natal, and Mitchell's Plain hospital in Western Cape.

b The characteristics of severe acute respiratory syndrome coronavirus 2-positive patients with SRI (cases) were compared with those of patients with ILI (controls).

c Hypertension data only available for those who indicated presence of condition under other underlying conditions.

d Evaluated underlying medical conditions included the following: asplenia or sickle cell anemia; chronic illness including lung, renal, liver, or cardiac disease; other immunocompromising conditions (excluding HIV), including organ transplant, primary immunodeficiency, immunotherapy, and malignancy; neurological disorders and burns.

e No/mild immune compromise CD4 count >200/mm^3^, severe immune compromise CD4 count ≤200/mm^3^.

*Any TB = TB at any time (past and current).

**Evaluated among women of childbearing age (15 years–49 years).

***Vaccination of healthcare workers (phase 1 of the vaccine program) with a single dose of the COVID-19 vaccine Jansen started on February 17, 2021 and phase 2 including the general public started on May 17, 2021. Full vaccination = 1 dose of COVID-19 vaccine Jansen or 2 doses of Pfizer-BioNTech COVID-19 Vaccine Comirnaty.

On multivariable analysis, comparing SARS-CoV-2-positive SRI cases with SARS-CoV-2-positive ILI cases, factors associated with increased risk of COVID-19-associated hospitalization included older age (25–44 years [aOR, 1.8; 95% CI, 1.1–2.9], 45–64 years [aOR, 6.8; 95% CI, 4.2–11.0], and ≥65 years [aOR, 26.6; 95% CI, 14.4–49.1] compared to 15–24 years), being of Black race (aOR, 3.3; 95% CI, 2.2–5.0), obesity (body mass index [BMI] ≥ 30 [aOR, 2.3; 95% CI, 1.4–3.9]), asthma (aOR, 3.5; 95% CI, 1.4–8.8), diabetes (aOR, 5.3; 95% CI, 3.1–9.3), previous or current tuberculosis (aOR, 12.8; 95% CI, 2.9–58.5), or HIV with CD4 ≥ 200/mm^3^ (aOR, 1.5; 95% CI, 1.1–2.2), and CD4 < 200/mm^3^ (aOR, 10.5; 95% CI, 5.1–21.6); being enrolled from KwaZulu-Natal (aOR, 2.6; 95% CI, 1.9–3.6) or Western Cape (aOR, 4.0; 95% CI, 2.6–6.0) compared to North West Province. Infection with Beta variant (aOR, 0.5; 95% CI, .3–.7) compared with Delta variant and being fully vaccinated versus not vaccinated/partially vaccinated (aOR, 0.1; 95% CI, .1–.3) were less likely to be associated with COVID-19-associated SRI hospitalization.

### Risk Factors for Coronavirus Disease 2019-Associated Mortality

During the study period, 7025 patients with SRI were enrolled at the surveillance hospital sites, 2282 (32.5%) of which tested positive for SARS-CoV-2. Of the 2255 COVID-19 patients with in-hospital outcome data, 387 (17.2%) died (Table 3). The median age of SARS-CoV-2 patients who died was 62.3 years (interquartile range, 52.1–69.5 years), 55.8% (216 of 387) were female, and 24.5% (87 of 355) were PWH. Older age (45–64 years [aOR, 2.6; 95% CI, 1.7–3.9] and ≥65 years [aOR, 4.7; 95% CI, 3.1–7.3] compared to 25–44 years) and admission for <3 days (aOR, 1.6; 95% CI, 1.1–2.2) compared to 3–7 days were associated with in-hospital mortality. Infection with Omicron (BA.1 and BA.2) (aOR, 0.3; 95% CI, .2–.6) compared with Delta variant was less likely to be associated with in-hospital mortality.

**Table 3.** Factors Associated With Mortality Among Patients Aged ≥15 Years Hospitalized With SRI Testing Positive for SARS-CoV-2, at 5 Sentinel Sites in South Africa, April 2020–March 2022, *n* = 2255

Characteristic	Case Fatality Ratio, *n*/*N* (%)	Odds Ratio (95% CI)	aOR (95% CI)
Age Groups	…	…	…
15–24 y	2/75 (2.7)	0.3 (.1–1.2)	0.3 (.1–1.6)
25–44 y	53/635 (8.4)	Reference	Reference
45–64 y	169/968 (17.5)	2.3 (1.7–3.2)	2.2 (1.6–3.2)
≥65 y	163/577 (28.3)	4.1 (2.9–5.7)	4.0 (2.8–5.8)
Sex			
Female	216/1389 (15.6)	Reference	Reference
Male	171/861 (19.9)	1.4 (1.1–1.7)	1.3 (1.0–1.6)
Race	…	…	…
Other	66/413 (15.6)	Reference	…
Black	321/1839 (17.5)	0.8 (.6–1.2)	…
Province	…	…	…
North West	62/509 (12.8)	0.8 (.5–1.2)	0.9 (.4–1.7)
Mpumalanga	68/275 (24.7)	1.9 (1.3–2.9)	2.0 (1.4–3.1)
KwaZulu-Natal	126/544 (23.2)	1.8 (1.2–2.5)	1.8 (1.2–2.8)
Western Cape	54/370 (14.6)	Reference	Reference
Gauteng	77/557 (13.8)	0.9 (.6–1.4)	1.3 (.9–2.0)
Wave vs out of wave	…	…	1.0 (.7–8.5)
Period since 1st case in South Africa (Months)	…	1.0 (1.0–1.0)	1.0 (.9–1.1)
Variant	…	…	…
Wuhan-Hu	66/445 (14.8)	0.6 (.4–.9)	0.8 (.4–1.7)
Alpha	4/19 (21.1)	1.1 (.3–3.3)	1.2 (.4–3.8)
Beta	95/437 (21.7)	0.9 (.7 –1.2)	1.0 (.6–1.5)
Delta	133/577 (23.1)	Reference	Reference
Omicron (BA.1 and BA.2)	17/197 (8.6)	0.3 (.2–.5)	0.3 (.2–.6)
20D (C.1.2)	5/14 (35.7)	2.0 (.7–6.3)	1.9 (.6–6.2)
Variant not assigned	40/380 (10.5)	0.4 (.3–.6)	0.5 (.3–.7)
Asthma	…		…
No	360/2066 (17.4)	Reference	…
Yes	12/76 (15.8)	0.9 (.5–1.6)	…
Diabetes	…		…
No	288/1777 (16.2)	Reference	…
Yes	99/472 (21.0)	1.3 (1.0–1.7)	…
Obesity (BMI ≥30)	…		…
No	335/2010 (16.7)	Reference	…
Yes	52/238 (21.9)	1.3 (.9–1.8)	…
HIV status	…	…	…
Negative	268/1511 (17.6)	Reference	…
Positive (no/mild immune suppression)^c^	27/218 (12.4)	0.6 (.4–1.0)	…
Positive (severe immune suppression)^c^	23/170 (13.5)	0.8 (.5–1.3)	…
Hypertension^a^	…	…	…
No	164/1055 (15.6)	Reference	…
Yes	162/757 (21.4)	1.5 (1.2–1.9)	…
Tuberculosis	…	…	…
No TB	379/2174 (17.4)	Reference	…
Any TB*	8/81 (9.9)	0.5 (.2–1.0)	…
RSV infection	…	…	…
No	370/2177 (17.0)	Reference	…
Yes	2/9 (22.2)	1.3 (.3–6.5)	…
Other Underlying Condition^b^	…	…	…
No	318/1833 (17.4)	Reference	…
Yes	69/422 (16.4)	1.0 (.8–1.3)	…
Pregnant	…	…	…
No	215/1223 (17.6)	Reference	…
Yes	1/164 (0.6)	0.03 (.00–.2)	…
COVID-19 Vaccination Status**			
Not vaccinated/partially vaccinated	373/2160 (17.2)	Reference	Reference
Fully vaccinated	8/69 (11.6)	0.6 (.3–1.2)	0.8 (.3–1.8)
Unknown	6/26 (23.1)	1.2 (.6–4.0)	1.3 (.5–3.9)
Duration of Symptoms (d)			
0–4 d	174/1074 (15.8)	Reference	…
≥5	213/1157 (18.4)	1.1 (.9–1.4)	…
Duration of Hospitalization (d)			
<3	250/1357 (18.4)	1.6 (1.2–2.1)	1.7 (1.2–2.2)
3–7	358/3034 (11.8)	Reference	Reference
≥8	331/2522 (13.1)	1.0 (.7–1.2)	1.0 (.7–1.3)

Abbreviations: aOR, adjusted odds ratio; BMI, body mass index; CI, confidence interval; d, days; HIV, human immunodeficiency virus; ICU, intensive care unit; ILI, influenza-like illness; RSV, respiratory syncytial virus; SARS-CoV-2, severe acute respiratory syndrome coronavirus 2; RSI, severe respiratory illness; TB, tuberculosis; y, years.

a Hypertension was not collected as a standalone variable; patients reported it under other underlying conditions.

b Evaluated underlying medical conditions included the following: asplenia or sickle cell anemia; chronic illness including lung, renal, liver or cardiac disease; other immunocompromising conditions (excluding HIV), including organ transplant, primary immunodeficiency, immunotherapy, and malignancy; neurological disorders and burns.

c No/mild immune suppression = CD4 count >200/mm^3^, severe immune suppression = CD4 count ≤200/mm^3.^

*Any TB = TB at any time (past and current).

**Vaccination of healthcare workers (phase 1 of the vaccine program) with a single dose of the coronavirus disease 2019 (COVID-19) vaccine Jansen started on February 17, 2021 and phase 2 including the general public started on May 17, 2021. Full vaccination = 1 dose of COVID-19 vaccine Jansen or 2 doses of Pfizer-BioNTech COVID-19 Vaccine Comirnaty.

## DISCUSSION

Using data from enhanced routine sentinel surveillance, we provide data on risk factors for COVID-19-associated hospitalization and mortality among individuals aged 15 years and older in a high HIV and high tuberculosis prevalence setting. Older age was strongly associated with hospitalization and mortality. In addition to older age, prior or current tuberculosis and severe HIV immunosuppression were associated with increased risk of COVID-19-associated hospitalization. Our study showed that compared with the Delta variant, the Omicron variant (BA.1 and BA.2) was associated with reduced risk of mortality and Beta associated with decreased risk of hospitalization. Similar to other studies, older age and underlying conditions (diabetes, obesity, asthma, HIV) were associated with severe COVID-19 illness [18, 19].

Compared to other causes of respiratory illness, SARS-CoV-2 was more likely to affect older individuals and was less likely to affect PWH. Human immunodeficiency virus infection has been associated with increased risk of severe illness associated with influenza [20] and bacterial pathogens such as the pneumococcus [21]. The apparent negative association with SARS-CoV-2 detection among SRI cases is likely because non- or mildly immunocompromised HIV may be less of a risk factor for SRI due to SARS-CoV-2 compared to other respiratory pathogens in the comparison group.

In this study, we identified risk factors for COVID-19-associated hospitalization that were similar to what has been described by other studies, such as older age, asthma, diabetes mellitus, tuberculosis, and obesity (BMI ≥30) [5, 18, 19, 22, 23]. In addition, increasing level of HIV immune compromise was also associated with higher risk of COVID-19 hospitalization. In population studies conducted during the first wave of SARS-CoV-2 infections, researchers reported an increased risk of hospitalization or mortality among PWH compared to the general population [5, 19]. In a population cohort study covering the first wave of infections in the Western Cape Province of South Africa, researchers reported increased risk of mortality (adjusted hazard ratio [aHR], 2.14) among PWH compared to the general population, and that CD4 < 200 cells/μL during current admission was associated with mortality [19]. Similarly, in a population-based study in England, researchers reported that HIV infection was associated with higher risk of death due to COVID-19 (aHR, 2.90; 95% CI, 1.96–4.30). In a cohort study in South Africa, among cases hospitalized with COVID-19, PWH with a history of immune compromise (CD4 count <200 cells/μL) were more likely to die in-hospital than those with CD4 counts of ≥200 cells/μL. However, CD4 count was only available for 20% of individuals [24]. Similarly, a retrospective cohort study using routinely collected data showed that after adjusting for sex and age, SARS-CoV-2-positive PWH compared to HIV-uninfected had higher risk of death (hazard ratio, 2.9; 95% CI, 2.0–4.2) [5].

Our study used a novel approach, leveraging longstanding syndromic sentinel surveillance for influenza and systematically collected epidemiological and SARS-CoV-2 genomic data and comparing data from outpatients and inpatients. This enhanced syndromic sentinel surveillance reported similar trends to those reported by the 2 national surveillance systems used in South Africa in response to COVID-19, the national laboratory-based surveillance and data for COVID-19 (DATCOV) national active surveillance for COVID-19 hospitalization [2, 24]. The common trends included the timing of the 4 waves of infection, each dominated by a different variant for the period under surveillance, higher prevalence of SARS-CoV-2 infection among females, and individuals of Black race compared to other races and similar risk factors for severe illness including older age and underlying illness such as tuberculosis and HIV with severe immunocompromise. In addition, the dominant variants in the waves also corresponded to the national data. Because sentinel surveillance provided generally robust data for tracking the SARS-CoV-2 pandemic comparable to data from national surveillance, it may represent a viable surveillance approach in settings where national surveillance is not possible and may also be a sustainable option for moving beyond the pandemic phase when testing for SARS-CoV-2 is reduced. The World Health Organization is encouraging countries to use systematically collected surveillance data to monitor the SARS-CoV-2 pandemic, and the guidance on the end-to-end integration of influenza and COVID-19 sentinel surveillance has been published [25].

In our sentinel surveillance, 17.2% of hospitalized cases with laboratory-confirmed COVID-19 died, and in-hospital mortality was highest among COVID-19 patients aged 65 years and older. This was slightly lower than the 26.2% reported for public sector hospitals included in the DATCOV national surveillance [26], most likely due to the fact that our surveillance system required patients to sign consent for participation, and critically ill patients may therefore have been missed. For the mortality model, we were not able to show a significant association with some risk factors reported by other studies, such as tuberculosis, or some underlying conditions (obesity [BMI ≥ 30], asthma, hypertension, chronic cardiac, and pulmonary conditions), possibly because numbers enrolled were too small to detect an association. This lack of power, especially for mortality analysis, represents a potential weakness of the sentinel surveillance approach that requires individual patient consent when compared to national surveillance. However, pooled analysis across different countries may be valuable.

Our study showed that the Omicron variant, which emerged in November 2021 in South Africa [27, 28], was associated with lower risk of mortality compared with the Delta variant. Our findings are similar to other studies [29–32]. Results from an analysis using data from national case, hospitalizations, and genomic surveillance systems showed that compared with Delta variant infections, S gene target failure-infections (proxy for Omicron variant) had a significantly lower odds of severe disease (aOR, 0.3; 95% CI, 0.2–0.5) among hospitalized individuals in South Africa [29].

The strengths of our study include that data were collected systematically in both inpatient and outpatient populations using an existing surveillance platform, and we were able to compare cases presenting with mild disease to those who were admitted. In addition, data on SARS-CoV-2 lineage was available for a majority of positive cases.

Our study has some limitations. Our surveillance covers 5 of the 9 provinces, including 3 of the most populous provinces in South Africa, and data from this study may not be generalizable to the rest of the country, especially if transmission patterns vary across provinces. For example, the phylogeographic analysis suggests that Beta variant, which was the driver of the second wave in South Africa, emerged in early August in Eastern Cape Province (which is not covered by surveillance) [33], but this variant was not detected in our enhanced sentinel surveillance until the first week of November 2020. Our study only collected data on in-hospital mortality, and it is possible that patients could have died after discharge from hospital or after ILI consultation.

## CONCLUSIONS

We demonstrate that an existing sentinel surveillance system could be modified to monitor the COVID-19 pandemic and likely any future pandemics once community transmission has been established. We described risk factors associated with SARS-CoV-2-associated hospitalization and mortality in a setting of high HIV and tuberculosis prevalence. Active syndromic surveillance combining clinical, laboratory, and genomic data can be used to describe the epidemic timing, epidemiological characteristics of cases, early detection of variants of concern, and how these impact disease severity and outcomes. The association of severe HIV-associated immune compromise with increased hospitalization highlights the importance of increasing access to antiretroviral therapy, and this group should be prioritized for vaccination. In addition, the increased risk of hospitalization and mortality among individuals aged ≥65 years supports South Africa's Department of Health guidance of including individuals aged >60 years as the priority group for COVID-19 vaccination and booster doses [34].

## Supplementary Material

ofac578_Supplementary_Data

## Supplementary Data

Supplementary materials are available at *Open Forum Infectious Diseases* online. Consisting of data provided by the authors to benefit the reader, the posted materials are not copyedited and are the sole responsibility of the authors, so questions or comments should be addressed to the corresponding author.
