# Supplementary material for: Risk Factors for Severe Coronavirus Disease 2019 Among Human Immunodeficiency Virus-Infected and -Uninfected Individuals in South Africa, April 2020–March 2022: Data From Sentinel Surveillance
Source: Open Forum Infect Dis. 2022 Nov 2;9(12):ofac578. doi: 10.1093/ofid/ofac578 (PMC9772867; doi:10.1093/ofid/ofac578)
Supplement: ofac578_Supplementary_Data [file ofac578_supplementary_data.pdf]

Risk factors for severe COVID-19 among HIV-infected and-uninfected individuals in South Africa, 2020-2022 – data from sentinel surveillance

Authors: Sibongile Walaza[1,2], Stefano Tempia[1,2,3,5], Anne von Gottberg[1, 6], Nicole Wolter[1,6] Jinal N. Bhiman[1]; Amelia Buys[1], Daniel Amoako[1], Fahima Moosa[1,6], Mignon du Plessis[1,6], Jocelyn Moyes[1,2], Meredith L. McMorrow[4,5], Halima Dawood[7,8], Ebrahim Variava [9,10], Gary Reubenson[11], Jeremy Nel[10], Heather J Zar[12], Mvuyo Makhasi[1], Susan Meiring[2,13], Vanessa Quan[13], Cheryl Cohen[1,2]

Affiliation

[1]Center for Respiratory Diseases and Meningitis, National Institute for Communicable Diseases of the National Health Laboratory Service, Johannesburg, South Africa

[2]School of Public Health, Faculty of Health Sciences, University of the Witwatersrand, Johannesburg, South Africa

[3]MassGenics, Atlanta, Georgia, United States of America

[4]Influenza Program, National Center for Immunization and Respiratory Diseases, Centers for Disease Control and Prevention, Atlanta, Georgia, United States of America

[5]Influenza Program, Centers for Disease Control and Prevention, Pretoria, South Africa.

[6]School of Pathology, Faculty of Health Sciences, University of the Witwatersrand, Johannesburg, South Africa

[7]Department of Medicine, Greys Hospital, Pietermaritzburg, South Africa

[8]Caprisa, University of KwaZulu - Natal, Pietermaritzburg, South Africa

[9]Department of Medicine, Klerksdorp-Tshepong Hospital Complex, Klerksdorp, South Africa

[10]Department of Medicine, Faculty of Health Sciences, University of the Witwatersrand, Johannesburg, South Africa

[11]Department of Paediatrics & Child Health, Faculty of Health Sciences, University of the Witwatersrand, Rahima Moosa Mother & Child Hospital, Johannesburg South Africa

[12]Department of Paediatrics, Red cross war Memorial hospital, Cape Town, South Africa

[13]Divison of Public Health Surveillance and Response, National Institute for Communicable Diseases of the National Health Laboratory Service, Johannesburg, South Africa

Background

The COVID-19 vaccine Jansen® booster was added on 24 December 2021. [1]. From 21 February 2022, individuals aged ≥ 18 years who had received the 1st dose of COVID-19 vaccine Jansen® were eligible for a COVID-19 vaccine Jansen® booster shot ≥60 days after first dose. From February 2022, individuals aged ≥18 years who had received a first dose of Pfizer vaccine were eligible to receive the second dose ≥21 days after first dose (a change from 42 previously recommended). From 14 March 2022, individuals who had received one booster following primary vaccination with COVID-19 vaccine Jansen® were eligible for an additional booster dose (COVID-19 Vaccine Comirnaty® OR COVID-19 vaccine Jansen®) ≥ 90 days from the first booster dose. Individuals who had received 2 doses (primary schedule) of Pfizer-BioNTech COVID-19 Vaccine Comirnaty® were eligible for a booster dose (Pfizer-BioNTech COVID-19 Vaccine Comirnaty® OR COVID-19 vaccine Jansen®) [2].

Methods

Laboratory Procedures

From 1 April 2020 – 28 February 2021, samples were tested for SARS-CoV-2 using the TIB MOLBIOL E gene assay (Roche Diagnostics)[3]. Influenza and RSV were detected using the Fast Track Diagnostics (FTD) Flu/HRSV kit (Siemens). From 1 March 2021, samples were tested using the Allplex™ SARS-CoV-2/Flu A/Flu B/RSV kit (Seegene, Seoul, South Korea). SARS-CoV-2 positivity was assigned if the PCR cycle threshold (C_t) value was <40 for ≥1 target (N, S or RdRp). Variants of concern (VOC) were determined by PCR as follows: from 1 April 2020 to 30 June 2021, SARS-CoV-2 samples were typed using the Allplex™ Variants I typing assay which detects Alpha and Beta/Gamma VOCs. In addition, samples collected from 1 January – 30 June 2021 were run on the Allplex™ Variants II assay which detects the Delta variant and differentiates Beta from Gamma. In addition to PCR, SARS-CoV-2 positive samples were sequenced to ascertain their lineage/clade. From July 2021, onwards, only sequencing was used to ascertain their lineage/clade.

For sequencing, briefly, RNA was extracted either manually or automatically in batches, using the QIAamp viral RNA mini kit (QIAGEN, CA, USA) or the Chemagic 360 using the CMG-1049 kit (PerkinElmer, MA, USA). Sequencing was performed with the amplicon-based next-generation sequencing approaches using the Illumina COVIDSeq protocol (Illumina Inc., CA, USA) or nCoV-2019 ARTIC network sequencing protocol v3 (https://artic.network/ncov-2019). Raw reads from Illumina sequencing were assembled using the Exatype NGS SARS-CoV-2 pipeline v1.6.1, (https://sars-cov-

2.exatype.com/). The resulting consensus sequence was further manually polished by considering and correcting indels in homopolymer regions that break the open reading frame (probably sequencing errors) using Aliview v1.27, (http://ormbunkar.se/aliview/)[4]. All assemblies determined to have acceptable quality (defined as having at least 1 000 000 reads and at least 50 % 10 X coverage) were deposited on GISAID (https://www.gisaid.org/)[5, 6]). Assembled genomes were assigned lineages using the 'Phylogenetic Assignment of Named Global Outbreak Lineages' (PANGOLIN) software suite (https://github.com/hCoV-2019/pangolin)[7]. The SARS-CoV-2 genomes were also classified using the clade classification proposed by NextStrain (https://nextstrain.org/)[8].

Ethical approval

In addition to the University of Witwatersrand Human Research Ethics Committee (HREC) approval, the ILI protocol was approved by the University of KwaZulu-Natal Human Biomedical Research Ethics Committee (BREC) reference BF 080/12 and the University of Cape Town Faculty of Health Science Human Research Ethics Committee (FHS HREC) reference 573/2018. In addition to the approval by the University of Witwatersrand HREC, the SRI protocol was approved by University of Cape Town FHS HREC reference 836/2014, and BREC reference M496/14.

Reference List

1. National Department of Health. Health on implementation of COVID-19 vaccine booster doses. Pretoria; 2021. Available at: Health on implementation of COVID-19 vaccine booster doses | South African Government (www.gov.za)
2. National Department of Health. Introduction of second booster dose after vaccination with COVID-19 vaccine Jansenn®. Pretoria; 2022. Available at :NATIONAL VACCINATION PROGRAMME CIRCULAR 4 OF 2022 - SA Corona Virus Online Portal2/
3. Corman VM, Landt O, Kaiser M, Molenkamp R, Meijer A, Chu DK, *et al*. Detection of 2019 novel coronavirus (2019-nCoV) by real-time RT-PCR. *Eurosurveillance* 2020,**25**:2000045.
4. Larsson A. AliView: a fast and lightweight alignment viewer and editor for large datasets. *Bioinformatics* 2014,**30**:3276-3278.
5. Elbe S, Buckland-Merrett G. Data, disease and diplomacy: GISAID's innovative contribution to global health. *Glob Chall* 2017,**1**:33-46.
6. Shu Y, McCauley J. GISAID: Global initiative on sharing all influenza data - from vision to reality. *Euro Surveill* 2017,**22**.
7. Rambaut A, Holmes EC, O'Toole Á, Hill V, McCrone JT, Ruis C, *et al*. A dynamic nomenclature proposal for SARS-CoV-2 lineages to assist genomic epidemiology. *Nature Microbiology* 2020,**5**:1403-1407.
8. Hadfield J, Megill C, Bell SM, Huddleston J, Potter B, Callender C, *et al*. Nextstrain: real-time tracking of pathogen evolution. *Bioinformatics* 2018,**34**:4121-4123.
